# Co-transplantation of Mesenchymal Stromal Cells and Induced Pluripotent Stem Cell-Derived Cardiomyocytes Improves Cardiac Function After Myocardial Damage

**DOI:** 10.3389/fcvm.2021.794690

**Published:** 2022-01-06

**Authors:** Klaus Neef, Florian Drey, Vera Lepperhof, Thorsten Wahlers, Jürgen Hescheler, Yeong-Hoon Choi, Tomo Šarić

**Affiliations:** ^1^Department of Cardiac and Thoracic Surgery, Heart Center, University of Cologne, Faculty of Medicine and University Hospital Cologne, Cologne, Germany; ^2^Institute for Neurophysiology, Center for Physiology and Pathophysiology, University of Cologne, Faculty of Medicine and University Hospital Cologne, Cologne, Germany; ^3^Clinic for Cardiac Surgery and Surgical Intensive Care Medicine, Kerckhoff Clinic Bad Nauheim, Kerckhoff Campus, Justus Liebig University Giessen, Giessen, Germany

**Keywords:** myocardial infarction, induced pluripotent stem cells, cardiomyocytes, mesenchymal stem cells, imaging

## Abstract

Induced pluripotent stem cell-derived cardiomyocytes (iPS-CMs) represent an attractive resource for cardiac regeneration. However, survival and functional integration of transplanted iPS-CM is poor and remains a major challenge for the development of effective therapies. We hypothesized that paracrine effects of co-transplanted mesenchymal stromal cells (MSCs) augment the retention and therapeutic efficacy of iPS-CM in a mouse model of myocardial infarction (MI). To test this, either iPS-CM, MSC, or both cell types were transplanted into the cryoinfarction border zone of syngeneic mice immediately after injury. Bioluminescence imaging (BLI) of iPS-CM did not confirm enhanced retention by co-application of MSC during the 28-day follow-up period. However, histological analyses of hearts 28 days after cell transplantation showed that MSC increased the fraction of animals with detectable iPS-CM by 2-fold. Cardiac MRI analyses showed that from day 14 after transplantation on, the animals that have received cells had a significantly higher left ventricular ejection fraction (LVEF) compared to the placebo group. There was no statistically significant difference in LVEF between animals transplanted only with iPS-CM or only with MSC. However, combined iPS-CM and MSC transplantation resulted in higher LVEF compared to transplantation of single-cell populations during the whole observation period. Histological analyses revealed that MSC increased the capillarization in the myocardium when transplanted alone or with iPS-CM and decreased the infarct scar area only when transplanted in combination with iPS-CM. These results indicate that co-transplantation of iPS-CM and MSC improves cardiac regeneration after cardiac damage, demonstrating the potential of combining multiple cell types for increasing the efficacy of future cardiac cell therapies.

## Introduction

Ischemic cardiomyopathy is currently the most frequent global cause of death (1). One of the main underlying pathophysiological issues in this disease is the limited intrinsic capacity of the human myocardium to regenerate after injury (2). The heart function is further diminished after the acute damage by unspecific inflammatory processes and adverse ventricular remodeling resulting in terminal heart failure (3). The recent progress in stem cell research has accelerated the field of regenerative medicine, often involving transplantation of cells to compensate for cell loss, rebuilding damaged tissue, and restoring the organ function (4–6). Different cell types have been investigated as treatment options for ischemic heart disease leading to variable results with respect to the therapeutic benefit (7).

Mechanistically, two groups of cell types with potential for regeneration can be distinguished. Firstly, the cells that indirectly support the endogenous regenerative capacities after transplantation and, secondly, the cells that functionally integrate into the damaged myocardium and directly contribute to the restoration of its pump function. The cells in the first group often belong to the category of adult stem cells, such as mesenchymal stromal cells (MSCs), which have been shown to act *via* paracrine factors reducing inflammation (8, 9), apoptosis (10), and adverse remodeling (11, 12) while enhancing vascularization and cell survival (13). MSCs have demonstrated safety and efficacy in preclinical and clinical trials of cardiac repair (12, 14–19), however, functional improvements have been limited and not sustained. A likely reason is the inability of MSC to restore lost contractility necessary for proper electromechanical heart function.

The cells in the second group are represented by electromechanically competent cardiomyocytes (CM) at various developmental stages. In pre-clinical studies, initially, the most promising results with respect to integration, electromechanical maturation, and functional improvement have been shown after intramyocardial transplantation of fetal and neonatal CM (20). However, ethical concerns and limited availability limit the prospects of clinical application of these cells. A promising alternative source of CM is pluripotent stem cells (PSCs), such as embryonic stem cells (ESCs) and induced pluripotent stem cells (iPSCs), which have the potential for essentially unlimited proliferation and capability to differentiate into the desired cell type. While the clinical translation of human ESC derivatives has been hindered by safety issues (21) and ethical concerns (22), the establishment of iPSC has opened new possibilities for research and regenerative medicine because iPSCs have the advantage of being autologous and able to generate derivatives with only limited immunological issues and ethical concerns (23).

Many studies have demonstrated that PSC-derived CMs have the potential to engraft and improve the performance of infarcted myocardium (24–29). However, these cells have only poor retention and very limited long-term survival after transplantation (30, 31), which represents one of the most formidable obstacles to clinical translation of stem cell-based cardiac regenerative therapies.

Previous studies have demonstrated that MSCs secrete anti-apoptotic factors that reduce apoptosis of isolated CM (32). We have shown that MSCs support the adhesion of iPSC-derived CM (iPS-CM) to non-contractile ventricular tissue slices and that factors secreted by MSCs improve electrical integration of iPS-CM into vital myocardial tissue *in vitro* (33) and counteract the effects of hypoxia on cultured iPS-CM (34). Therefore, we sought in this study to investigate whether MSCs are capable of augmenting the engraftment and reparative capacities of co-transplanted iPS-CM *in vivo* using a murine model of myocardial infarction (MI).

## Materials and Methods

### Generation of CM From Murine iPSC

**Figure 1:**
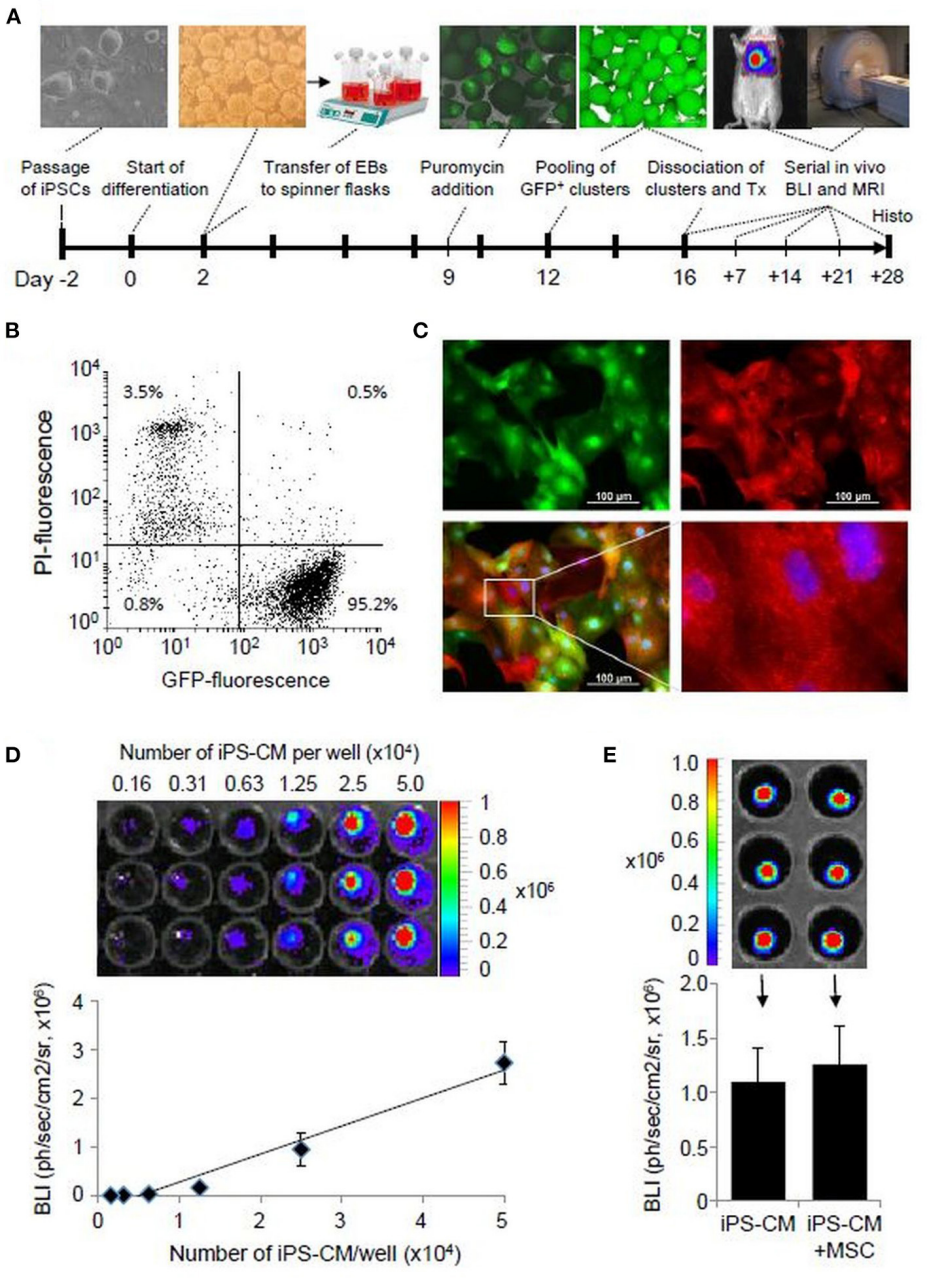
iPS-CM characterization. **(A)** Schematic diagram of cardiac iPSC differentiation and experimental plan. **(B)** Flow cytometric analysis of eGFP-positive puromycin selected FLuc-αPIG-iPS-CM at day 16 of differentiation (2 x 10^4^ events). PI-propidium iodide. **(C)** Immunofluorescence detection of eGFP (green) and α-actinin (red) in purified iPS-CM plated on fibronectin-coated dishes. Nuclei were stained with Hoechst. **(D)** Optical signal intensity of serial dilutions of 5.0 x 10^4^ iPS-CM expressing FLuc was measured in IVIS. The image in the upper panel shows a representative result of one serial dilution in triplicates. The lower panel demonstrates the linearity between cell dose and BLI of iPS-CM shown in the upper panel. The data are given as mean ± SD. **(E)** Determination of the BLI signal of 10^4^/well FLuc-αPIG-iPS-CM alone or mixed with the same number of MSC in IVIS. Data in the bar graph are shown as mean ± SD of two different batches of iPS-CM and MSC each measured in triplicates as shown in the representative IVIS image in the upper panel. The statistical significance of differences in groups was checked by the unpaired student's *t*-test. IPSC, induced pluripotent stem cells; eGFP, enhanced green fluorescent protein; CM, cardiomyocytes.

Cardiomyocytes were generated from transgenic murine iPSC line pUbC-FLuc-αPIG (clone C3) that expresses luciferase under the control of a constitutive ubiquitin C promoter (pUbC) and puromycin resistance and enhanced green fluorescent protein (eGFP) genes under the control of a cardio-specific α-myosin-heavy-chain promoter. The generation, characterization, and cardiac differentiation of this cell line have been described elsewhere (35). The differentiation of iPSC is induced by embryoid body (EB) formation as illustrated in Figure 1A using Iscove's Modified Dulbecco's Medium (IMDM) supplemented with 20% fetal bovine serum (FBS), 1 × non-essential amino acids, 0.1 mM β-mercaptoethanol (all from Life Technologies, Carlsbad, CA, USA), and 50 μg/ml ascorbic acid (Wako Chemicals USA Inc., Richmond, VA, USA) in non-adherent cell culture dishes on a shaker. After 2 days, 3 × 10^4^ EB were transferred to spinner flasks (Integra, Hudson, NH, USA) containing 200 ml of differentiation medium. On day 9 of differentiation, 8 μg/ml puromycin (Invivogen, San Diego, CA, USA) was added to the media for CM selection. On day 12, the CM clusters were transferred to a 10 cm cell culture dish and cultured on a shaker until day 16 when they were dissociated with 0.25 g/l trypsin and 0.2 g/l ethylenediaminetetraacetic acid (EDTA, both Life Technologies) containing 5 U/ml DNAse (Sigma-Aldrich, St. Louis, MO, USA) and used for transplantation experiments.

### Isolation and Expansion of Bone Marrow-Derived MSC

Mesenchymal stromal cells were isolated and expanded as previously described (36). Briefly, the bone marrow cells were flushed from long bones of 8-week old C57BL/6 mice with phosphate-buffered saline (PBS, Life Technologies) and cultured in mMSC media (PAN Biotech, Aidenach, Germany), supplemented with 2.5 ng/ml human basic fibroblast growth factor (bFGF, R&D Systems, Wiesbaden, Germany), 100 U/ml penicillin, and 100 μg/ml streptomycin (both Life Technologies). A proliferative, morphologically homogenous MSC population was established after continuous culture and passaged at 80% confluence using trypsin/EDTA for 6–8 weeks. The adipogenic, osteogenic, and hondrogenic differentiation potential of isolated MSCs was assessed as described previously (36).

### Magnetic Labeling of MSC

The MSCs were labeled with paramagnetic microspheres (diameter: 0.9 μm; composition: polystyrene with 62% (w/w) iron oxide; fluorescent label: Flash Red; Bangs Laboratories, Fishers, IN, USA), serving as MRI contrast agent and histological marker as described before (36). Briefly, the cells were incubated overnight with 11.2 × 10^6^ microspheres/cm^2^ cell culture surface in MSC cell culture media. The MSCs were co-labeled with the fluorescent vital dye Vybrant DiI-CM (Life Technologies) following the instructions of the manufacturer to identify the transplanted MSC-containing microspheres in the histological analyses.

### Immunocytochemistry

Single iPS-CMs from trypsinized cardiac clusters were plated on fibronectin-coated (Tebu-Bio, Offenbach, Germany) culture dishes (μ-Dish 35 mm, high; Ibidi, Martinsried, Germany). Adherent iPS-CMs were fixed with 4% paraformaldehyde (PFA; Polysciences, Eppelheim, Germany) and permeabilized with 0.5 M ammonium chloride (Roth, Karlsruhe, Germany) and 0.25% Triton X-100 (Sigma-Aldrich). The cells were then incubated overnight with α-actinin antibodies (1:800, Sigma-Aldrich) and visualized with species-matched secondary Alexa Fluor 555-conjugated antibodies (Life Technologies). The nuclei were counterstained with Hoechst 33342 (Life Technologies).

### Flow Cytometry

Induced pluripotent stem cell-derived-CMs were analyzed using a FACScan instrument (BD, Franklin Lakes, NJ, USA). The dead cells were identified with propidium iodide (PI, BD). Cell debris and dead cells were gated out, and the remaining cells were quantified for expression of eGFP using CellQuest v2.0 software (BD). The MSC surface markers were analyzed using the following fluorescently labeled antibodies: anti-CD29 (phycoerythrin, PE), anti-CD44 (biotin, anti-biotin-PE-Vio770), anti-CD90.2 (VioBlue), anti CD105 (allophycocyanin, APC), anti-Sca-1 (fluorescein isothiocyanate), and anti-CD11b (APC-Vio770) and fluorophore-matched isotype controls (all Miltenyi Biotec, Bergisch Gladbach, Germany). Cells were analyzed using a MACSquant flow cytometer and MACSquantify software (version: 2.4, both Miltenyi Biotec) with a 3% threshold (isotype control vs. specific antibody).

### Animal Care

All animal experiments described in this study were approved by the Landesamt für Natur, Umwelt und Verbraucherschutz NRW (LANUV, Recklinghausen, Germany; Permit Number: 8.87-50.10.37.09.161) and conformed to the Directive 2010/63/EU of the European Parliament. Efforts were made to minimize the suffering of animals.

### Animal Model/Intramyocardial Transplantation

A myocardial injury was induced by cryo-infarction in 8–10 weeks old male C57BL/6 mice as described previously (36). Briefly, mice were anesthetized with 3% isoflurane (Baxter, Unterschleißheim, Germany) in the absence of muscle relaxants, placed onto a heating plate warmed to 38°C, intubated and ventilated with a mixture of nitrous oxide and oxygen (1:1) and 1.25% isoflurane at a rate of 130 heaves per minute with a volume of 0.5–0.8 ml. After performing a skin incision, the muscles were loosened, and the thoracic cavity was opened by inserting a retractor into the intercostal space between the third and fourth rib. The heart was exposed, the left ventricular myocardium was cryoinjured, and 5 μl of cell suspension was injected into the single periinfarct region using a 25 μl Hamilton syringe (Model 702 RN SYR, Hamilton, Bonaduz, CH) and a 27G needle. Postoperative analgesia was provided by subcutaneous administration of Tramadol at 15 mg/kg after the extubation and at 1 mg/ml in the drinking water for 4 additional days (Gruenenthal, Aachen, Germany). Immediately after infarct induction, the mice were randomly assigned to the following four experimental groups to receive 1) iPS-CM, 2) MSC, 3) iPS-CM + MSC, and 4) vehicle (PBS) injection (placebo group). For each cell type, 5 × 10^5^ cells in a total volume of 5 μl PBS were injected into a single site of the peri-infarct region with a 25 μl syringe (Hamilton, Bonaduz, Switzerland) and a 27G cannula (BD). The mortality rate of animals was in the range between 10 and 20% and did not significantly differ between experimental groups. The great majority of mice died shortly after surgical intervention, most likely due to detrimental effects resulting from infarction.

### Bioluminescence Imaging

D-luciferin (Caliper, Hopkinton, MA, USA) was administered i.p. at a concentration of 300 mg/kg body weight per mice, which were then anesthetized with 2.5% isoflurane (Baxter, Unterschleissheim, Germany) for image acquisition (IVIS200 system, Caliper) as described by us earlier (35). The BLI acquisition time was 60 seconds with binning set to maximum. The image acquisition was performed on the day of cell transplantation, days 1 and 3, and weekly up to 28 days after transplantation. The BLI signal was quantified with the Living Image 3D software (version: 2.5.1., Caliper).

### Magnetic Resonance Imaging

Transplanted magnetically labeled MSCs were identified, and cardiac function was assessed as described previously (36). Serial MRI scans were performed weekly for 28 days after infarct induction and cell transplantation. ECG-gated sagittal scans of six slices covering six cardiac phases were used to localize microsphere labeled MSC in long-axis images of the left ventricle. For cardiac function assessment, the ECG-gated transversal images of 6 slices with 12 cardiac phases of the left ventricle were acquired between the end-systolic and end-diastolic states. The mice were anesthetized during MRI scanning with 1.25% isoflurane (1 l/min O_2_), and normothermia was maintained with a heating system in the solenoid coil. The location of MSC was determined by analyzing the long-axis images for signal voids caused by the paramagnetic microspheres within the left ventricular wall and were saved as image files using the visualization software DICOM viewer R2.5 v1.1 (Philips Amsterdam, The Netherlands). As a parameter of cardiac function, the left ventricular ejection fraction (LVEF) was calculated from end-systolic and end-diastolic volumes calculated from semi-automatically assessed endocardial and epicardial contours using image analysis software Segment v1.8 (Medviso, Lund, Sweden).

### Immunohistochemistry and Histology

Mice were euthanized after the final MRI scan, the hearts were excised, flushed with PBS, and cryo-preserved using Tissue-Tek O.C.T. (Sakura Finetek, Staufen, Germany). The hearts were cryo-sectioned (10 μm), and the presence of iPS-CM was determined by screening for eGFP fluorescence, and MSCs were detected by screening for Vybrant DiI-CM and flash red fluorescence of microspheres. Cryo-sections were fixed with 4% PFA and incubated with anti-α-actinin (1:800, Sigma-Aldrich), anti-connexin-43 (1:750, Sigma-Aldrich), anti-eGFP (1:200, Life Technologies), or anti-caveolin-1 (1:800, Acris, Herford, Germany). Species-specific fluorescent secondary antibodies (Alexa Flour 488, 594, or 647, Life Technologies) were used for the detection of primary antibodies. Fibrotic areas were identified by Masson's Trichrome staining (Roth) following the instructions of the manufacturer. Images were acquired using an Eclipse Ti-U microscope and NIS Elements v3.22 software (Nikon, Düsseldorf, Germany). Entire mouse hearts were transversally cryo-sectioned. For quantitative analysis of fibrotic scar, every tenth section was stained with Masson's Trichrome. The size of the fibrotic scar was assessed by measuring the outside (epicradial) length of the fibrotic area (blue staining) per transversal histological section. These lengths were multiplied with the long-axis distance between sections (100 μm) and added up as per heart, as a representation of the size of the cryo-injury induced fibrotic scar area.

### Statistical Analyses

Data are presented as means with SE for continuous variables. Differences in continuous variables in two groups were examined using student's t-test. The one-way ANOVA test was used to compare values between more than two groups. When the one-way ANOVA test was significant, group differences were compared using the *post-hoc* Tukey-Kramer test. Statistical analyses were performed using InStat (GraphPad Software, San Diego, CA, USA). The statistical significance was defined as *P* < 0.05.

## Results

### Generation of Reporter iPS-CM

The differentiation of murine iPSC line pUbC-FLuc-αPIG and puromycin selection generated a homogeneous population of spontaneously beating clusters, which contained more than 95% viable and eGFP expressing iPS-CM and yielded an average of 2.5 CM per iPSC initially used (Figures 1A,B). Immunocytochemical analysis with α-actinin antibodies confirmed iPS-CM purity and showed that these cells exhibit intra-cellular sarcomeric organization typical for heart cells (Figure 1C). The BLI signal of purified iPS-CM was detectable *in vitro* with high sensitivity, correlated in a linear fashion with cell dose (Figure 1D), and was not affected by the addition of MSC (Figure 1E). The minimal amount of cells that can be reliably detected by BLI *in vivo* was 5 × 10^4^ transplanted iPS-CM [(35) and our unpublished data]. These data demonstrate that iPS-CMs generated in this protocol are suitable for quantitative monitoring of their retention *in vivo* using BLI.

### Isolation and Labeling of MSC

**Figure 2:**
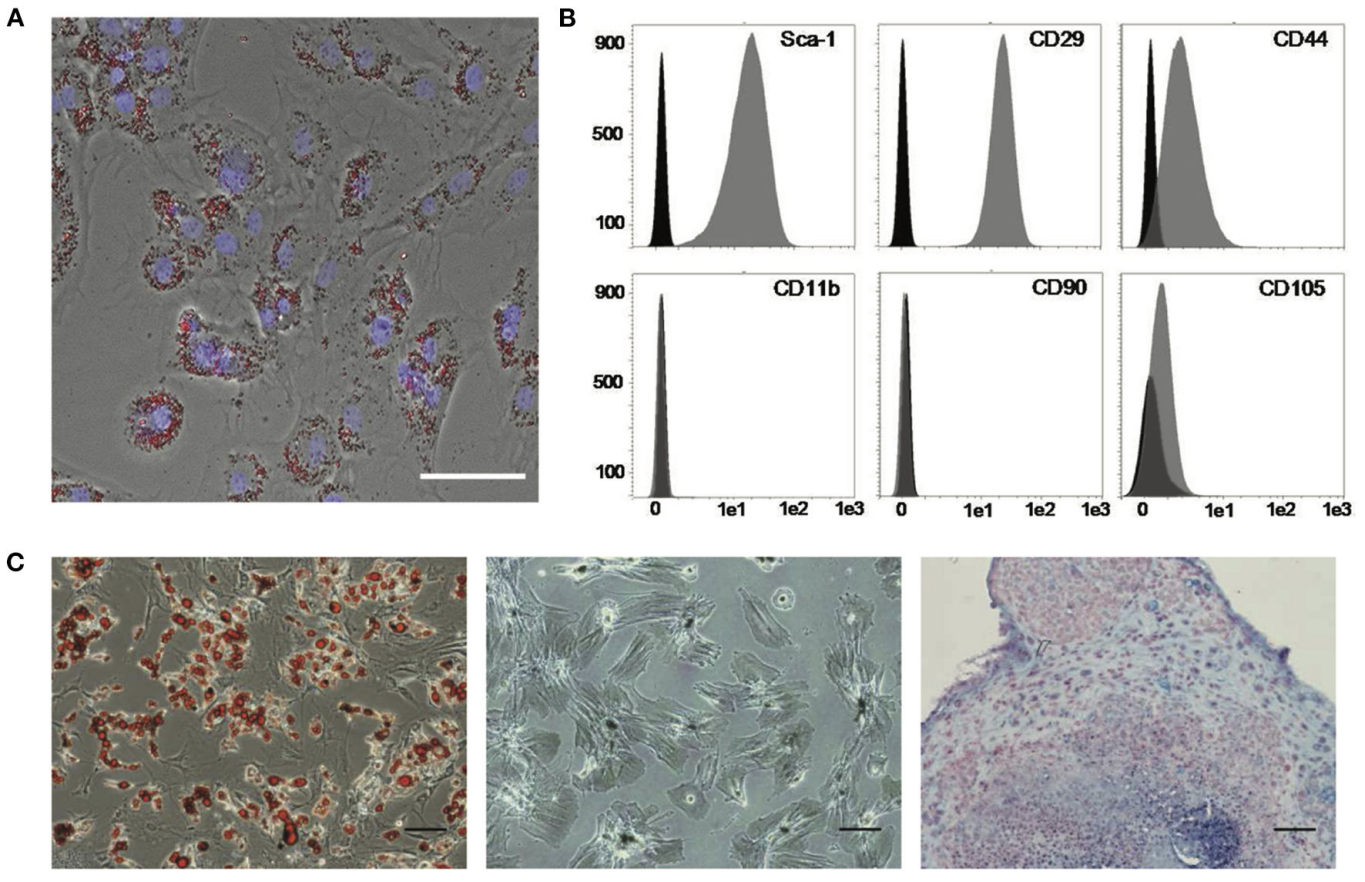
Labeling and characterization of MSC. **(A)** Labeling of MSC with paramagnetic and fluorescent microspheres (red). Nuclei were counterstained with DAPI (blue). Scale bar: 50 μm. **(B)** Flow cytometric analysis of MSC. Representative histograms for indicated surface markers used for validation of MSC identity are shown in gray. Black histograms show control measurements with isotype-matched unspecific antibodies. **(C)** Multi-lineage differentiation potential of MSC into adipogenic, osteogenic, and chondrogenic derivatives (from left). Cells were stained for lipid vesicles (red, left panel), intracellular calcium deposits (black, middle panel), and hyaluronic acid in extracellular matrix (blue, right panel), indicative of respective cell types. Scale bars: 100 μm. MSC, mesenchymal stromal cells.

After 6–8 weeks in culture and expansion for additional 3–4 passages, MSC freshly isolated from the bone marrow of C57BL/6 mice exhibited a morphologically homogeneous, proliferative population of spindle-shaped cells. Overnight incubation of MSC with microspheres resulted in almost complete labeling of the MSC population (Figure 2A) without interfering with MSC defining features, as reported previously (36). The analysis of cell-type defining surface markers confirmed the expression of CD29 (99.95 ± 0.02%), CD44 (74.91 ± 24.11%), and Sca-1 (90.88 ± 13.60%), whereas CD11b, CD90, and CD105 were not expressed (Figure 2B). Cultivation of MSCs in selective media confirmed that they possess adipogenic, osteogenic, and chondrogenic differentiation potential (Figure 2C).

### BLI Does Not Show Enhanced Retention of iPS-CM by Co-transplanted MSC

**Figure 3:**
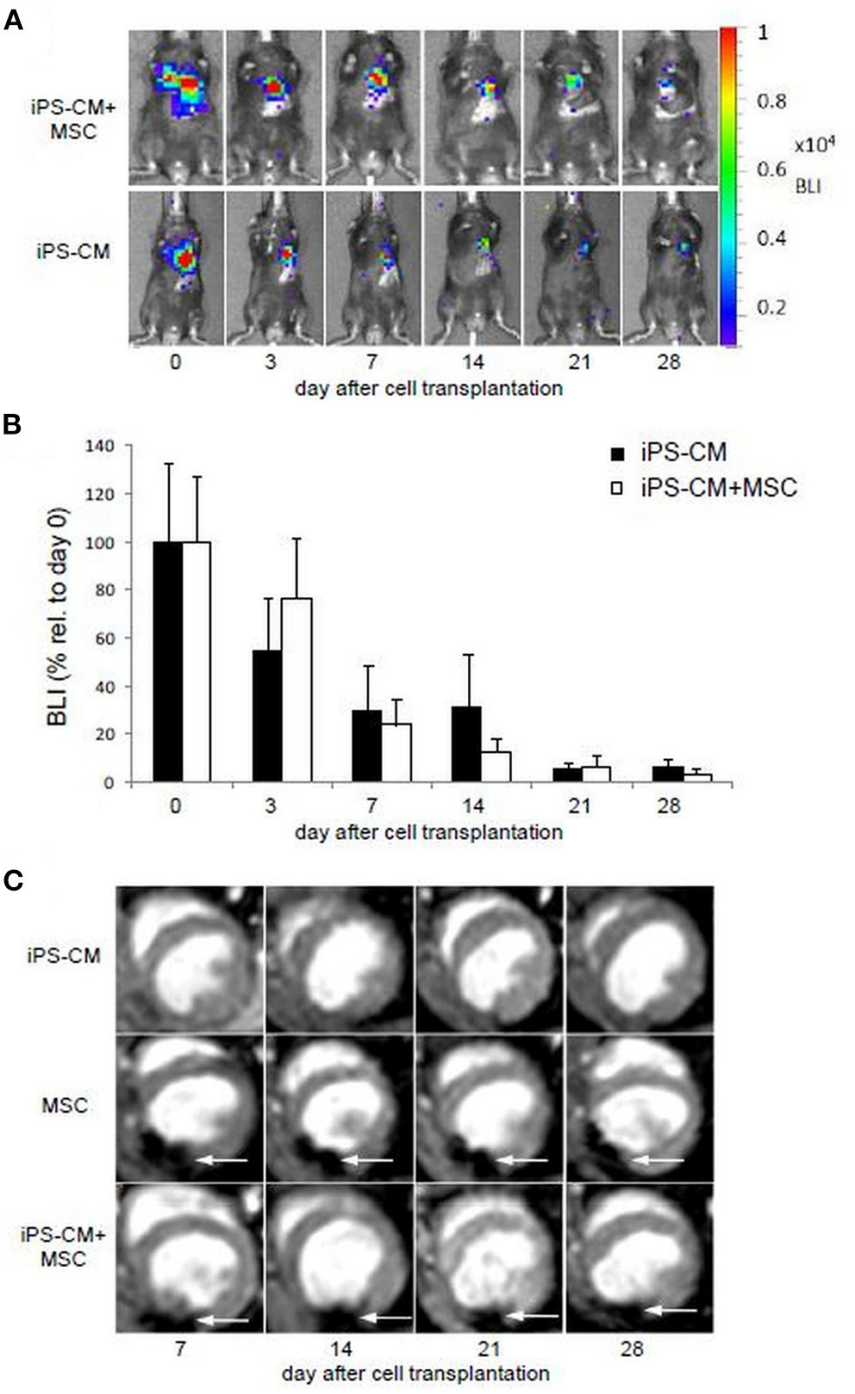
Cell tracking and assessment of cardiac function after induction of MI and application of different cell therapy approaches. **(A)** BLI measurements showing representative animals from the group having received iPS-CM and iPS-CM+MSC over the course of 28 days. **(B)** Quantitative analysis of BLI in animals transplanted with iPS-CM (*n* = 9) or iPS-CM+MSC (*n* = 9). Data are given as mean ± SD of percentages of values relative to BLI on day 0 of cell transplantation. **(C)** MRI reveals the static location of the microsphere labeled MSC at the injection site over the course of 28 days in representative animals from the MSC and iPS-CM+MSC groups. Animals from the iPS-CM group showed no signal from injected cells. White arrows indicate signal voids caused by labeled MSC. BLI, Bioluminescence imaging; CM, cardiomyocytes; MSC, mesenchymal stromal cells; iPS, induced pluripotent stem cell.

To determine if the retention and survival of transplanted iPS-CM in cryoinjured heart can be enhanced by combined application with MSC, we compared the BLI signal intensity in animals transplanted with 5 × 10^5^ iPS-CM alone or in combination with 5 × 10^5^ magnetically labeled MSC in a total volume of 5 μl PBS during the follow-up period of 28 days after transplantation (Figures 3A,B). BLI signals were only detected in the heart region and were not disseminated to other areas of the body of transplanted animals. On day 3, after transplantation, the BLI signal in animals that received only iPS-CM significantly decreased to 50.2 ± 26.3% (*P* < 0.001) compared to the signal measured immediately after transplantation, while the intensity of the BLI signal in the iPS-CM+MSC group decreased to a lesser extent (62.4 ± 26.5%; *p* > 0.05 compared to day 0) but this difference did not reach statistical significance (Figure 3B). On day 7, the BLI signal dropped from basal value on day 0 to 23.8 ± 17.9% and 33.1 ± 21.8% (both *P* < 0.001 compared to day 0) in iPS-CM+MSC and iPS-CM groups, respectively and continued to decrease steadily to values below 7% on day 28 (iPS-CM: 6.2 ± 5.1%; iPS-CM+MSC: 2.7 ± 3.1%). These data indicate that co-administration of MSC did not significantly affect the retention of viable luciferase-expressing iPS-CM in the infarcted hearts during the 28-day observation period.

### MSCs Increase the Number of Animals in Which iPS-CMs Are Histologically Detectable

**Figure 4:**
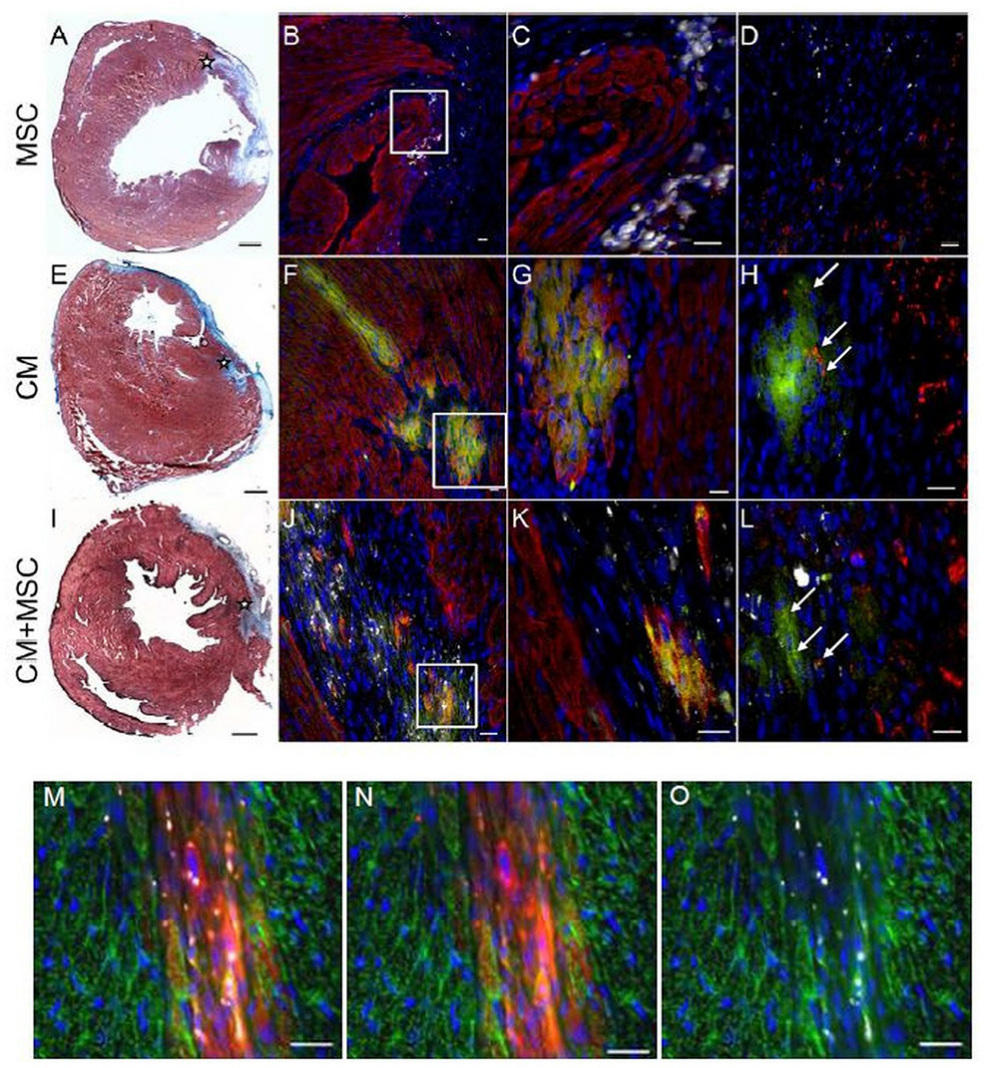
Immunohistochemical detection of transplanted cells in host myocardium. Representative photomicrographs from three experimental groups 4 weeks after intramyocardial injections of either MSC **(A–D)**, iPS-CM **(E–H)**, or iPS-CM+MSC **(I–L)**. **(A,E,I)** *Masson's Trichrome* stainings of transverse sections of hearts 4 weeks after operations with fibrotic scar tissue stained blue. The injection site in the peri-infarct region is marked with an asterisk. Scale bars: 500 μm. **(B,F,J)** Immunohistological stainings of sections containing transplanted cells 4 weeks after operations. eGFP (green), α-actinin (red), microspheres (white), nuclei (blue). Scale bars: 20 μm. **(C,G,K)** Magnified view of areas in white boxes in images at left. Scale bars: 20 μm. **(D,H,L)** Immunohistological stainings of the same area in neighboring sections of sections shown in images at left. eGFP (green), connexin 43 (red), microspheres (white), nuclei (blue). Scale bars: 20 μm. **(M,N,O)** Immunohistological stainings of sections from the MSC group with grafted MSC showing co-localization of internalized microspheres (white) with membrane stain Vybrant DiI-CM (red). α-actinin (green), nuclei (blue). Scale bars: 20 μm. eGFP, enhanced green fluorescent protein; CM, cardiomyocytes; MSC, mesenchymal stromal cells.

Proper intramyocardial localization of magnetically labeled MSC in both experimental groups was assessed by weekly ECG-triggered cardiac MRI measurements. The scans of placebo or iPS-CM-transplanted animals did not show any signal voids in the myocardium (Figure 3C). However, analyses of animals transplanted with MSC and iPS-CM+MSC revealed signal voids over the course of 28 days, which did not change their location or intensity during this time period (Figure 3C). Since the signal voids detected by MRI may not originate from viable MSC but also from microparticles released from dead MSC and deposited within the tissue or macrophages, the presence of cells in myocardial tissue sections was assessed by fluorescence microscopy on day 28 after transplantation. These analyses confirmed the survival of at least a fraction of transplanted MSC in the peri-infarction region in all animals of MSC (100%; 20/20) and iPS-CM+MSC (100%; 22/22) groups (Figures 4A–D,I–L). Transplanted MSC could be identified by fluorescence signals emitted by the membrane stain Vybrant-DiI-CM, which co-localized in the same areas that contained microspheres (Figures 4M–O). Engrafted iPS-CMs were identified in tissue sections as isolated patches of eGFP-positive cells (Figures 4E–H,I–L), which expressed cardiac α-actinin organized in sarcomeric structures (Figures 4G,K) and connexin 43 in few defined areas (Figures 4H,L, white arrows). Interestingly, small areas of eGFP-positive iPS-CM were detected histologically in 86.4% (19/22) of animals that were co-transplanted with iPS-CM and MSC, while only 47.4% (9/19) of animals contained GFP-positive cells in myocardial tissue sections in the iPS-CM only group. These findings indicate that co-administration of MSC did not significantly affect the BLI signal of viable luciferase-expressing iPS-CM in the infarcted heart during the 28-day observation period but increased the number of animals in which iPS-CM could be microscopically detected, which was most likely due to the inability of the BLI method to detect small enhancement of CM survival in individual animals.

### Co-transplantation of iPS-CM and MSC Improves Recovery of Heart Function

**Figure 5:**
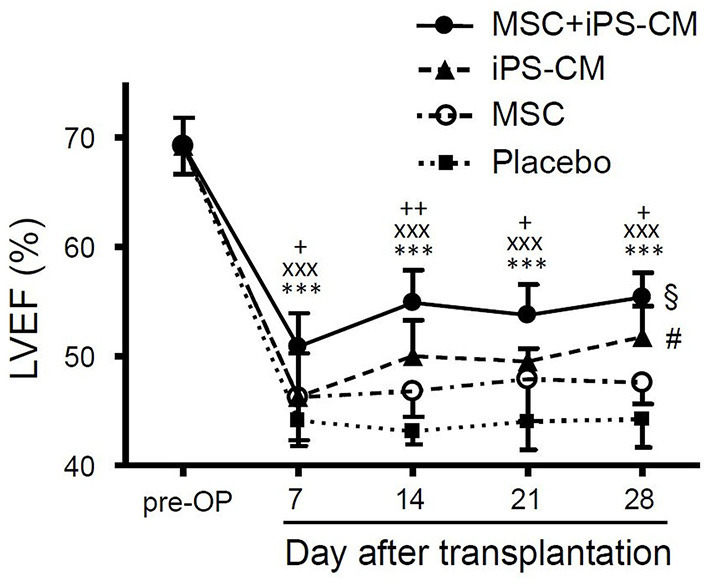
Assessment of cardiac function from weekly cardiac MRI scans after induction of MI. LVEF was determined in healthy animals pre-operatively and at weekly intervals after MI and cell transplantation in groups that received PBS (*n* = 9), MSC (*n* = 9), iPS-CM (*n* = 8), or iPS-CM+MSC (*n* = 8). Statistically significant results for iPS-CM+MSC group: ***P < 0.001 vs. placebo, ^xxx^*P* < 0.001 vs. MSC, ^+/++^*P* < 0.05/0.01 vs. iPS-CM; ^§^*P* < 0.05: iPS-CM+MSC on day 1 vs. iPS-CM+MSC on day 7; ^#^*P* < 0.05: for iPS-CM on day 1 vs. iPS-CM on day 7 (all one-way ANOVA with the Tukey-Kramer *post-hoc* test). CM, cardiomyocytes; MSC, mesenchymal stromal cells; iPS, Induced pluripotent stem cell; LVEF, left ventricular ejection fraction.

To assess the changes in heart function after MI and cell transplantation, the LVEF in all experimental groups was determined by weekly MRI measurements over the course of 28 days (Table 1). On day 7, after the induction of MI, a significant decrease in LVEF from 69.2 ± 2.6% (*n* = 5) in healthy animals to 44.1 ± 2.3% (*n* = 9) in vehicle-transplanted animals was observed, and LVEF in the latter group did not change significantly during the entire follow-up period of 28 days (Figure 5). However, co-delivery of iPS-CM and MSC resulted in a significant increase in LVEF in 8 mice assessed by MRI compared to vehicle-injected controls and single cell-type recipients at all time-points measured (Figure 5, Table 1). Animals that received MSC alone or iPS-CM alone demonstrated better function than animals in the placebo group on days 14, 21, and 28 after transplantation, but the difference in LVEF between these single cell-type recipients was significant only on postoperative day 28 with iPS-CM exerting a stronger therapeutic effect (MSC: 47.6 ± 1.9, *n* = 9; iPS-CM: 51.8 ± 2.8%, *n* = 8; *P* < 0.01). Interestingly, intragroup comparisons revealed that only the animals that received iPS-CM alone or iPS-CM combined with MSC showed significant improvement of the LVEF during the 28 day follow-up period (iPS-CM: 46.3 ± 4.0% on day 7 vs. 51.8 ± 2.8% on day 28, *P* < 0.01; iPS-CM+MSC: 50.9 ± 3.0% on day 7 vs. 55.4 ± 2.3% on day 28, *P* < 0.001; Figure 5). Taken together, dual cell therapy with iPS-CM and MSC resulted in earlier, more efficient, and more sustained functional recovery of acutely injured myocardium than treatment with either cell type alone.

**Table 1:** Statistical analysis of LVEF results from cardiac MRI measurements by ANOVA and *post hoc* Tukey-Kramer multi-group comparison tests.

		**Placebo** **(*n* = 9)**	**MSC** **(*n* = 9)**	**iPS-CM** **(*n* = 8)**	**MSC +** **iPS-CM** **(*n* = 8)**
day 7	Placebo	44.1 ± 2.3^*^	ns	ns	*P* < 0.01
	MSC	ns	**46.2 ± 3.9**	ns	*P* < 0.05
	iPS-CM	ns	ns	**46.3 ± 4.0**	*P* < 0.05
	iPS-CM+MSC	*P* < 0.01	*P* < 0.05	*P* < 0.05	**50.9 ± 3.0**
day 14	Placebo	**43.1 ± 1.2**	*P* < 0.05	*P* < 0.001	*P* < 0.001
	MSC	*P* < 0.05	**46.8 ± 2.3**	ns	*P* < 0.001
	iPS-CM	*P* < 0.001	ns	**50.0 ± 3.3**	*P* < 0.01
	iPS-CM+MSC	*P* < 0.001	*P* < 0.001	*P* < 0.01	**54.9 ± 3.0**
day 21	Placebo	**44.0 ± 2.5**	*P* < 0.05	*P* < 0.01	*P* < 0.001
	MSC	*P* < 0.05	**47.9 ± 3.8**	ns	*P* < 0.001
	iPS-CM	*P* < 0.01	ns	**49.5 ± 1.2**	*P* < 0.05
	iPS-CM+MSC	*P* < 0.001	*P* < 0.001	*P* < 0.05	**53.8 ± 2.8**
day 28	Placebo	**44.2 ± 2.6**	*P* < 0.05	*P* < 0.001	*P* < 0.001
	MSC	*P* < 0.05	**47.6 ± 1.9**	*P* < 0.01	*P* < 0.001
	iPS-CM	*P* < 0.001	*P* < 0.01	**51.8 ± 2.8**	*P* < 0.05
	iPS-CM+MSC	*P* < 0.001	*P* < 0.001	*P* < 0.05	**55.4 ± 2.3**

* *Values in bold at intersections of each experimental group represent LVEF for this group and the corresponding time point expressed as mean ± SD. ns, non-significant (p > 0.05)*.

### Co-transplantation of iPS-CM and MSC Improves Myocardial Capillarization and Reduces Scar Size

**Figure 6:**
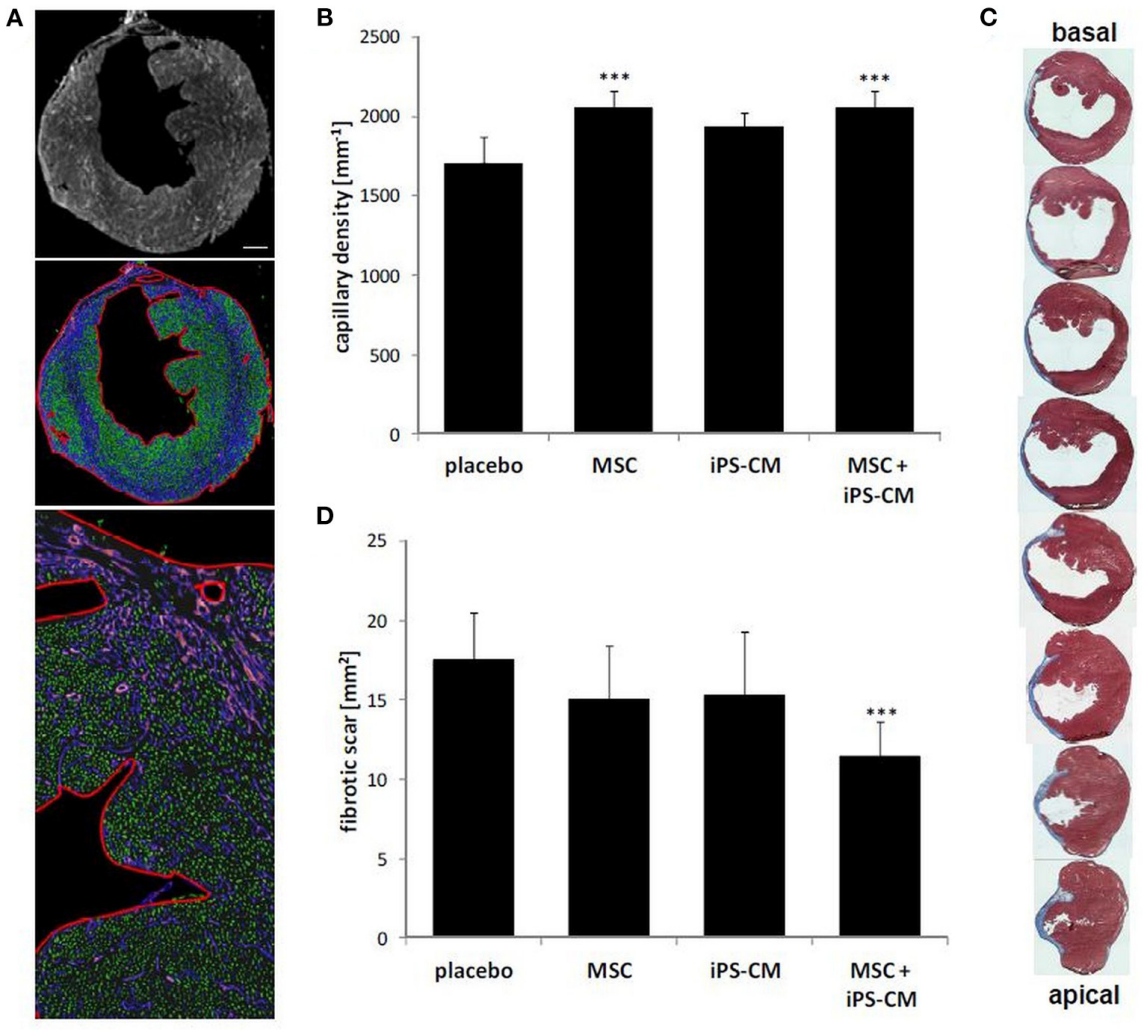
Assessment of scar area and capillary density in hearts 4 weeks after MI and cell transplantation. **(A)** Immunohistological staining of transversal heart sections for the endothelial-specific marker caveolin-1 (white in the top panel; violet in middle and lower panels). Capillary density was determined by image analysis software (Nikon Elements NIS) according to the size and circularity of fluorescents objects (marked in green in middle and lower panel) within defined regions of the myocardium (outlined in red in the middle and lower panel). Scale bar: 500 μm. **(B)** Quantitative analysis of capillary density 4 weeks after cell transplantation in each experimental group. ***P < 0.001 vs. placebo. **(C)** Serial Masson's Trichrome stained sections (blue: fibrotic area) of hearts 4 weeks after induction of MI from basal to apical infarction border. Gaps between displayed sections are 100 μm. **(D)** Quantitative analysis of infarction scar expansion based on Masson's Trichrome stained sections in indicated experimental groups. ***P < 0.001 vs. placebo (all one-way ANOVA with the Tukey-Kramer *post-hoc* test).

Further histological and immunohistochemical analyses of myocardial tissue sections obtained 28 days after operations revealed significantly increased myocardial capillary density in the MSC group (2,051.2 ± 108.1 capillaries/mm^2^; *P* < 0.001) and the iPS-CM+MSC group (2,050.9 ± 109.2/mm^2^; *P* < 0.001) compared to the placebo group (1,704.4 ± 173.0/mm^2^; Figures 6A,B). Additionally, the analysis of the fibrotic area in left-ventricular wall infarction scars revealed a significant reduction of scar size only in the iPS-CM+MSC group (11.5 ± 2.1 mm^2^ vs. placebo: 17.5 ± 3.0 mm^2^; *P* < 0.001; Figures 6C,D). There was no statistically significant difference in scar size in other inter-group comparisons. These data suggest that enhancement of capillarization in the infracted myocardium mostly depends on MSC-specific factors, whereas reducing scar size requires co-transplantation of both cell types.

## Discussion

Many studies demonstrated that PSC-derived CMs have the capacity to engraft and build functional myocardium and improve cardiac performance in different animal models of MI (24, 37, 38). However, the maximal therapeutic potential of this cell population has not yet been fully exploited, because of the poor engraftment and survival of exogenous CM at the site of injection (30, 35). Therefore, improving these parameters is crucial for achieving the best possible myocardial regeneration after MI. The present study was designed to investigate if MSC can augment the retention of co-transplanted iPS-CM in damaged myocardium and if combined cell therapy can improve the ventricular pump function. Our *in vivo* analyses showed that MSC could not significantly increase the retention of iPS-CM in the peri-infarct the region of myocardium. Therefore, the observed improvement of LVEF in animals that received combination therapy compared to placebo and single-cell populations most likely resulted from isolated or partially overlapping but additive effects of each single cell type. Although the exact mechanism responsible for therapeutic effects observed in our study remains to be elucidated, MSCs most likely conferred their beneficial effects by paracrine factors acting on vascularization and tissue remodeling (39, 40) and iPS-CMs contributed to improved heart function both by secreted factors (41, 42) and their electromechanical properties (24, 26).

Our study quantitatively assesses the effect of co-transplanted MSC on survival and therapeutic effect of iPS-CM by serially and non-invasively monitoring the retention kinetics of viable luciferase-expressing CM using BLI and cardiac function via MRI. Among previous studies comparing the effects of single and combined cell therapies in animal models of MI (41, 43–49), only Kearns-Jonker et al. investigated the added benefits of MSCs and ESC-derived CMs in a rat model of MI but they did not quantify cell retention (45). Ye and coworkers used quantitative PCR (qPCR) to assess cell retention in their study comparing therapeutic effects of human iPS-CM alone or in combination with iPSC-derived endothelial cells and smooth muscle cells in a pig model of ischemia/reperfusion injury (41). They showed that significantly more cells survived in the co-transplantation group after 4 weeks, however to a still generally low extent (4.1 vs. 3.2%). Although the qPCR analysis did not account for viability and type of the detected cells, these data emphasize the effects of combining multiple cell types to modulate and at best improve cardiac cell therapy approaches. This is in agreement with our observation that MSC did not increase the survival of iPS-CM to an extent detectable by BLI. There are many factors influencing the efficacy of cell retention after transplantation, in particular, mechanical washout immediately after injection (30). Additionally, functional iPS-CM engraftment could be limited or prevented by suboptimal cell dose and cell formulation, improper timing of transplantations, or inability of the engrafted dose of MSC to mitigate the adverse effects of the acutely infarcted tissue microenvironment. Furthermore, iPS-CM might lack structural features necessary for stable attachment in the host tissue, as previously shown for murine ESC-derived CM (50).

Therefore, to increase the efficacy of cardiac cell-based therapies significant effort must be directed toward improving the survival and functional integration of transplanted cells into the host tissue. To this end, more efficient cell delivery tools and methodologies and optimized therapeutic formulations of defined cell types combined with biomaterials and cardioprotective factors should be developed and tested (51). The importance of this approach was demonstrated by Ye et al. who showed that a two-fold improvement of cell survival at 4 weeks after transplantation can be achieved by transplanting iPS-CM, EC, and SMC through an insulin-like growth factor 1 (IGF-1)-secreting epicardial fibrin patch, suggesting that prevention of mechanical cell loss and cardioprotective factors play a more important role in enhancing cell retention than simultaneous co-delivery of different cell populations (41). Further improvements of cell retention could be achieved with pharmacological (small molecules) or genetic agents (exosomes, microRNA) employed locally or systemically to stimulate endogenous repair mechanisms and act beneficially via immuno-modulation, anti-inflammation, and neovascularization.

Despite very poor cell retention, we found that from week 2 after transplantation and onwards the animals in all groups that received cells had a significantly higher LVEF compared to the placebo group, regardless of only MSC, only iPS-CM, or both cell types combined were transplanted. There was no statistically significant difference in LVEF between animals transplanted with iPS-CM alone or MSC alone. However, co-transplantation of both cell types led to a higher LVEF compared to transplantation of single-cell populations at each time point analyzed. These findings are in agreement with previous studies demonstrating in general the higher efficacy of cell combinations compared to single-cell treatments, independently of which cell combinations were examined (41, 43–48, 52, 53). However, the therapeutic effects of cell combinations in individual studies were rather heterogeneous. Kearns-Jonker et al. showed that co-transplantation of human MSC and ESC-derived CM was more beneficial than transplantation of CM alone in inducing the expression of genes in host cells that encode for factors that promote cardiac repairs, such as hepatocyte growth factor or IGF-1 (45). Notably, in contrast to our findings, synergistic effects from co-transplantation with respect to cardiac function (LVEF) have not been observed. Williams et al. found that cardiac performance was preferentially improved after MI in animals receiving a combination of MSC and CSC but the LVEF was restored to baseline level in all cell therapy groups irrespective of cell type injected (47). Interestingly, Ye and co-workers reported no improvement of LVEF after transplantation of human iPS-CM alone or in combination with iPSC-derived EC and SMC compared to the vehicle control group (41). This parameter was increased at 4 weeks after co-transplantation only when a fibrin patch containing IGF-1 was applied together with all three cell populations.

In a study comparing the therapeutic effect of fetal CM and MSC administered alone into ischemic myocardium of mice with MI, fetal CM led to significantly smaller infarcts, less adverse remodeling, better cardiac function, and longer survival compared to transplantation of MSC, indicating that CM might be required to restore myocardial function, in contrast to non-contractile MSC (20). Recent studies underline the hypothesis of synergistic effects of co-transplantation of iPS-CM and MSC for post-MI repair (52). Interestingly, Yoshida et al. showed elegantly that co-administered MSCs reduce immune rejection of allogeneic iPS-CM, however, not performing intramyocardial injections, but using a subcutaneous setting to increase control, emphasizing MSC-mediated immunomodulatory effects leading to enhanced survival of transplanted iPS-CM (54). These promising findings are reflected and emphasized by pre-clinical studies showing synergistic effects of allogenic cardiac progenitor cells and MSC for reduction of infarct scar sizes and functional parameters in large animal models (53) and, very recently, clinical studies showing positive effects from transplantation of c-kit^+^ cardiac cells together with MSC in patients with heart failure from ischemic heart disease (55, 56). In our study, the infarct scar area was not significantly decreased by either iPS-CM or MSC but only when both cell types were co-administered. Furthermore, capillary density was increased only when MSCs were transplanted alone or together with iPS-CM. Discrepant results obtained in our and in different studies discussed above may be explained by differences in animal species, cell types, infarction models, and other disparate variables, which need to be standardized for more meaningful comparisons.

Taken together, our data demonstrate that iPS-CMs are essential for the recovery of cardiac function after MI and that MSCs further improve their therapeutic effect without enhancing CM retention. While both cell types were required for attenuating the fibrotic infarction area, MSCs alone were sufficient for stimulating angiogenesis in the myocardium. These results provide support for the concept that application of complimentary cell populations has higher regenerative potential than a single cell population, and that enhancement of cell retention required for the enhanced therapeutic outcome may not be achieved by transplanting different mixtures of cells but rather by pursuing other strategies, such as those that prevent immediate cell loss after injection and counteract the death of transplanted cells later after transplantation. Since the beneficial effects do not correlate with the number of functionally engrafted cells, the underlying mechanisms still remain to be elucidated.

## Data Availability Statement

The raw data supporting the conclusions of this article will be made available by the authors, without undue reservation.

## Ethics Statement

The animal study was reviewed and approved by Landesamt für Natur, Umwelt und Verbraucherschutz NRW (LANUV), Recklinghausen, Germany, Permit Number: 8.87-50.10.37.09.161.

## Author Contributions

KN: conception and design, financial support, collection and assembly of data, data analysis and interpretation, manuscript writing, and final approval of manuscript. FD and VL: collection and assembly of data, data analysis and interpretation, and manuscript writing. TW and JH: financial support and final approval of manuscript. Y-HC: conception and design, financial support, and final approval of manuscript. TŠ: conception and design, financial support, collection and assembly of data, data analysis and interpretation, manuscript writing, and final approval of manuscript. All authors contributed to the article and approved the submitted version.

## Funding

This study was supported by grants from the Federal Ministry for Education and Research to TŠ, Y-HC, and JH (Grant No. 01GN0947) and from the Else-Kröner-Fresenius Stiftung to TŠ and KN (Grant No. A93/2008). Further funding was provided by the Maria Pesch Stiftung and Köln-Fortune Program to TŠ.

## Conflict of Interest

The authors declare that the research was conducted in the absence of any commercial or financial relationships that could be construed as a potential conflict of interest.

## Publisher's Note

All claims expressed in this article are solely those of the authors and do not necessarily represent those of their affiliated organizations, or those of the publisher, the editors and the reviewers. Any product that may be evaluated in this article, or claim that may be made by its manufacturer, is not guaranteed or endorsed by the publisher.
